# Study protocol for FUTURES: Testing a web-based reproductive health education program for adolescent and young adult males with sickle cell disease

**DOI:** 10.1371/journal.pone.0289039

**Published:** 2023-07-26

**Authors:** Zachary A. Colton, Charis J. Stanek, Sophia M. Liles, Christian Baker, Toyetta Barnard-Kirk, Peter Chan, Ben McCorkle, Gwendolyn P. Quinn, Yvette Shen, Charleen I. Theroux, Susan E. Creary, Leena Nahata

**Affiliations:** 1 The Abigail Wexner Research Institute at Nationwide Children’s Hospital, Columbus, Ohio, United States of America; 2 The Ohio State University, Columbus, OH, United States of America; 3 Nationwide Children’s Hospital, Columbus, Ohio, United States of America; 4 New York University School of Medicine, New York, New York, United States of America; University of Illinois at Chicago, UNITED STATES

## Abstract

Individuals with sickle cell disease are increasingly surviving into adulthood, many of whom have interest in future biological parenthood. Reproductive health knowledge is low among adolescent and young adult males and their caregivers. Their understanding of these topics is needed to optimize their reproductive health outcomes. As such, through collaboration with a community advisory board (adolescents and young adults with sickle cell disease and mothers of adolescent and young adult males with sickle cell disease) and digital design team, we developed a web-based sickle cell disease-focused reproductive health program entitled FUTURES to address these knowledge gaps. For phase I of this two phase feasibility and acceptability study, adolescent and young adult males and their caregivers will complete a pre- and post-program reproductive health knowledge and attitudes questionnaire to assess change in knowledge. In phase II, after learning about fertility testing as part of the FUTURES curriculum, adolescent and young adult male participants are given the option to pursue testing. The two-phase study aims to: 1) develop and test the feasibility, acceptability, and efficacy of a reproductive health web-based educational program at increasing reproductive health knowledge in male adolescent and young adult males with sickle cell disease and their caregivers, and 2) assess feasibility of fertility testing. The long-term goal is to improve reproductive and psychosocial outcomes among adolescent and young adult males with sickle cell disease.

## Introduction

An estimated 100,000 individuals within the United States are living with sickle cell disease (SCD); many of these individuals are underserved racial minorities with limited health literacy [1–3]. SCD is characterized by life-long, debilitating symptoms that lead to significant decreases in quality of life. Advances in medical treatments have led to substantial increases in symptom management and survival rates, with current life expectancy estimates ranging from 38 to 54 years [1–3]. Given the growing number of adults with SCD, there is an increasing need to examine and address the long-term effects of SCD and its treatments, including impacts on reproductive health outcomes. Further, multiple calls for initiatives to address gaps in SCD health-related knowledge have been made [4–6].

There are various implications that SCD and its treatments have on adolescent and young adult (AYA) males’ reproductive health. Initial studies found that hypogonadism, defined as low sperm concentration and testosterone production, can occur in up to 25% of males with SCD [7–9]. Additionally, a recent study found that 91% of adult males with SCD and 100% of those treated with hydroxyurea, the primary treatment for SCD, had abnormal semen parameters, raising concerns that SCD and its treatments may increase the risk for fertility impairment [10]. This is notable as studies with other populations at risk for fertility impairment, such as male childhood cancer survivors, show that parenthood is a high priority and that infertility in adulthood is associated with psychosocial distress [11]. Finally, discussions about the genetic inheritance of SCD and contraception are also critical since AYA are at a developmental stage associated with romantic relationship development and sexual behaviors [12]. Research suggests low rates of contraception use and high rates of unplanned pregnancies among the SCD population [13, 14]. Despite increasing awareness about the wide spectrum of reproductive health implications of SCD, educational resources for these AYA remain limited, and those that exist exclude fertility-related content and/or do not target adolescents [15–17].

A recent pilot study by the current research team examining reproductive health knowledge in male AYA with SCD and their caregivers showed that 85% of AYA expressed interest in having a biological child in the future [18]. Additionally, a large proportion of AYA (70% and 65% respectively) and caregivers (80% and 53% respectively) expressed unfamiliarity with the purpose and process of fertility testing, and a desire for more information about how SCD and its treatments may impact reproductive health [18]. Lastly, 50% of AYA and 40% of caregivers reported never having received information about the potential infertility risks associated with SCD and its treatments from a provider [18]. These findings highlight the need for efficacious, evidence-based educational tools addressing reproductive health topics for AYA with SCD.

Developing culturally appropriate and accessible education tools on reproductive health is especially important for this population, given the many barriers to care and knowledge that AYA with SCD face. Some of these obstacles include limited economic resources, lack of transportation to appointments, and low health literacy [3, 6, 19, 20]. Additionally, minoritized populations impacted by SCD often have poor patient-provider communication resulting in patients feeling ambivalent towards receiving medical care and unprepared for medical decision making [21]. AYA also report being uncomfortable discussing sexual health care with their provider because of the sensitivity of these topics, and physicians report lacking the SCD-specific knowledge and confidence to facilitate these conversations [22, 23]. Preliminary research has supported feasibility and efficacy of a web-based format for delivering sexual and reproductive health education in SCD populations [24, 25].

### Aims

Given these gaps in clinical care, the primary aim of this two-phase study is to develop and test a culturally appropriate, developmentally appropriate reproductive health education program entitled FUTURES (Fertility Education to Understand Reproductive Health in Sickle Cell Disease; © 2023, Nationwide Children’s Hospital) for male AYA with SCD and their caregivers. The secondary aim is to explore feasibility, facilitators, and barriers to fertility testing in male AYA with SCD. Our primary hypothesis is that both AYA and caregiver reproductive health knowledge about SCD will significantly increase following completion of the FUTURES program.

## Methods

### Ethics

This study was approved by the Institutional Review Board (#STUDY00002516) on 5/17/2022. All participants (AYA between 18 and 22 years of age and their caregivers) will provide written informed consent and AYA younger than 18 will provide written assent. Relevant data will be made available at the conclusion of this study upon request.

### Design

This study is a single site, two phase cross-sectional study. In phase I, upon recruitment by research staff, participants will complete several baseline questionnaires. AYA and caregivers will then watch FUTURES on an electronic device and complete additional follow-up questionnaires assessing health literacy and post-program knowledge and acceptability of the program. If AYA are ≥18 years of age or if they are younger and have a caregiver who also participates, they will then be informed about the second phase of the study. We will review all enrolled AYAs’ electronic medical records (EMR) and abstract data on their clinical course, including treatments and SCD complications.

Phase II will occur approximately two weeks later, with the research team contacting eligible AYA to determine interest in fertility testing. If they express interest, the study team will send an order for fertility testing to a local testing site who will coordinate a testing appointment directly with the AYA. If testing is completed, the hematologist on the study team will provide the AYA and their caregiver (if the AYA <18 years old) the results. All participants who are eligible for phase II will be invited to complete a brief interview to inquire about facilitators or barriers to testing (dependent on testing decision).

### Setting, recruitment, and participants

Potential participants at a large, Midwest pediatric academic medical center will be screened from the clinical SCD database. We aim to recruit 40 AYA and approximately 20 of their caregivers (anticipating one caregiver for each participant <18 years of age). Recruitment will either occur at a scheduled hematology or transfusion visit, while the AYA is hospitalized, or remotely via telephone. Remote recruitment will occur if the participant requests to do the study remotely when approached in-person or if they do not have a visit scheduled during the study timeframe.

Phase I participants will be AYA aged 14 to 22 years with SCD and their caregivers (AYA under 18 requiring caregiver consent). Inclusion criteria for AYA are as follows: having SCD (any genotype), receiving hematology care at the medical center, proficiency in English, and the cognitive ability to complete survey measures. Exclusion criteria include a history of bone marrow transplant and involvement in the prior pilot study [18]. Inclusion criteria for caregivers are as follows: having legal guardianship of an AYA enrolled in the study, proficiency in English, and the cognitive ability to complete survey measures. Phase II participants will be the AYAs who completed Phase I and are ≥18 years of age and younger AYA who also have a caregiver who participates in Phase I.

### Measures

**Fig 1:**
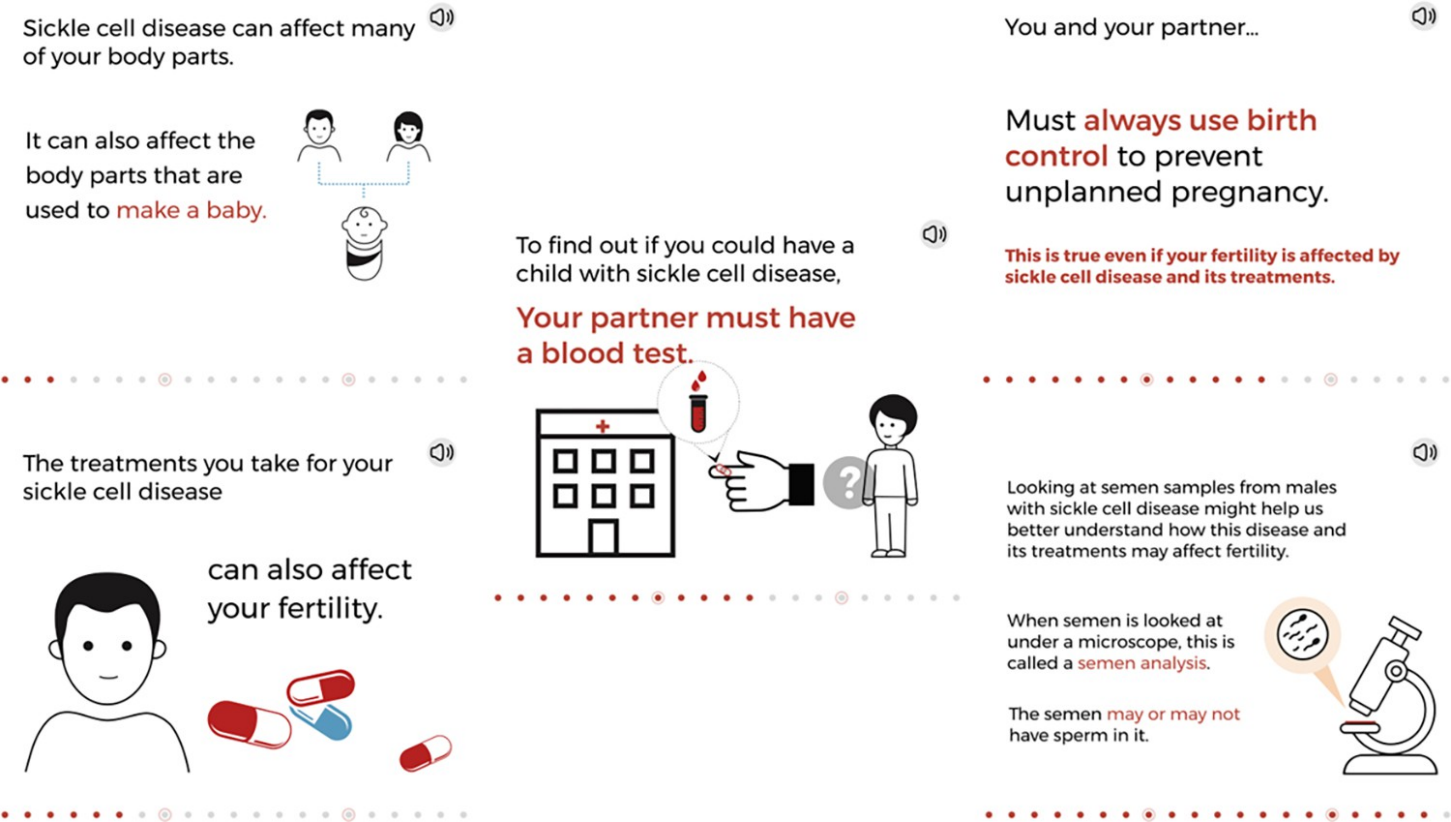
The knowledge objective slides. © 2023, Nationwide Children’s Hospital.

Phase I will include questionnaires that assess demographic information, parenthood goals, health literacy (Newest Vital Signs, NVS; [26]) and reproductive health knowledge pertaining to SCD (Fertility Knowledge and Attitudes Questionnaire, FKAQ) (Table 1). This questionnaire was created by the research team based on a previous survey [18] and then pilot tested for clarity with a male AYA with SCD and their caregiver. It will also include a satisfaction survey adapted from a previous study by the research team [27] for FUTURES (Fig 1). For phase II, participants will complete a facilitators or barriers interview (Table 2), depending on if the AYA completes fertility testing.

**Table 1:** Phase I measure descriptions.

Measure	Description
**Demographics**	All participants will self-report demographic and background characteristics (e.g., age, race, ethnicity, relationship status).
**Fertility Knowledge and Attitudes Questionnaire (FKAQ) [18]**	A Likert-scale survey assessing knowledge about the fertility implications of SCD and its associated treatments, where they receive their fertility-related knowledge about SCD and its treatments, as well as knowledge about fertility testing and how it is completed. The AYA version will also ask the AYA about their parenthood goals, while the caregiver version will ask about their son’s parenthood goals and their desire to have a biological grandchild.
**Newest Vital Signs (NVS) [26]**	A health literacy tool validated for use with adolescents and adults, regardless of educational attainment [26, 28] Participants are given the nutrition label for a container of ice cream and asked six questions about said information. Scores range from 0–6, and participants with scores <4 will be considered to have limited health literacy.
**Satisfaction Survey [27]**	Participants will be asked their opinions about the effectiveness of the educational program’s visual/audio materials, method used to administer the education (individual, online), its language and content, its cultural appropriateness, the length of the program, the helpfulness of the program, how it impacted how they think about their (their son’s) SCD and fertility. Open-ended questions will also be included to gather feedback on how to improve and broaden the program.

**Table 2:** Phase II measure descriptions.

Measure	Description
**Facilitators Interview**	We will examine facilitators of fertility testing (e.g., transportation provided, fee waiver, coordination), what factors they considered, and who influenced their decision to pursue fertility testing.
**Questions**	1) “How did you come to the decision to complete fertility testing?” a. What resources offered (e.g., transportation, money, coordinating from research team), if any, influenced your decision? b. What other factors did you consider in your decision to complete fertility testing? c. Who, if anyone, influenced your decision to complete fertility testing?
	2) “Is there anything else you would like to share with us about your decision or experience in this study that we have not yet discussed?
**Barriers Interview**	We will identify remaining barriers to fertility testing, ask about who influenced their decision, and ask what the research team could have done to make the option of fertility testing easier.
**Questions**	1) How did you come to the decision to opt-out of the fertility testing option? a. What were the obstacles you faced to completing fertility testing? b. What other factors did you consider in your decision not to complete fertility testing? c. Who, if anyone, influenced your decision not to complete fertility testing?
	2) What, if anything could we have done to make this option of fertility testing easier for you and your family?
	3) Is there anything else you would like to share with us about your decision or experience in this study that we have not yet discussed?

#### Intervention

*FUTURES study team*. To create a reproductive health education program that was comprehensive, culturally and developmentally appropriate, and engaging, a multidisciplinary team was assembled. This team included clinicians with expertise in reproductive health and SCD (a pediatric endocrinologist and hematologist), clinical research coordinators, a community advisory board (CAB), and a visual communication design team from a university. A CAB was included based on a lack of community-based SCD research and prior research indicating the importance of understanding the target population’s perspectives and knowledge base to improve project outcomes [29]. CAB members included two mothers of male AYA with SCD and three AYA males (aged 19–21 years) with SCD. CAB members were invited by the clinicians based on their prior successful engagement in research and interest in promoting SCD knowledge in the community. The design team consisted of an English professor, two visual communication design professors, and an undergraduate student majoring in design. The three professors on the design team had previously worked with the hematologist on a web-based, health-related educational program about sickle cell trait [27, 30]. With their academic background in writing and visual rhetoric, they focused on creating effective and engaging digital health-related material through a user-centered design approach.

*FUTURES development*. Initially, data from the pilot study [18] and recent literature [31–36] were used to draft the program’s knowledge objectives. Subsequently, three, two-hour meetings were held with the research team in which CAB members provided overall feedback and suggestions regarding the drafted knowledge objectives, ideal program features (game-like, intuitive technology, self-guided), and the comprehensibility of the content. Additionally, CAB members indicated that FUTURES would ideally be delivered in conjunction with a clinic visit and would last no longer than 15 minutes to ensure sustained attention.

**Fig 2:**
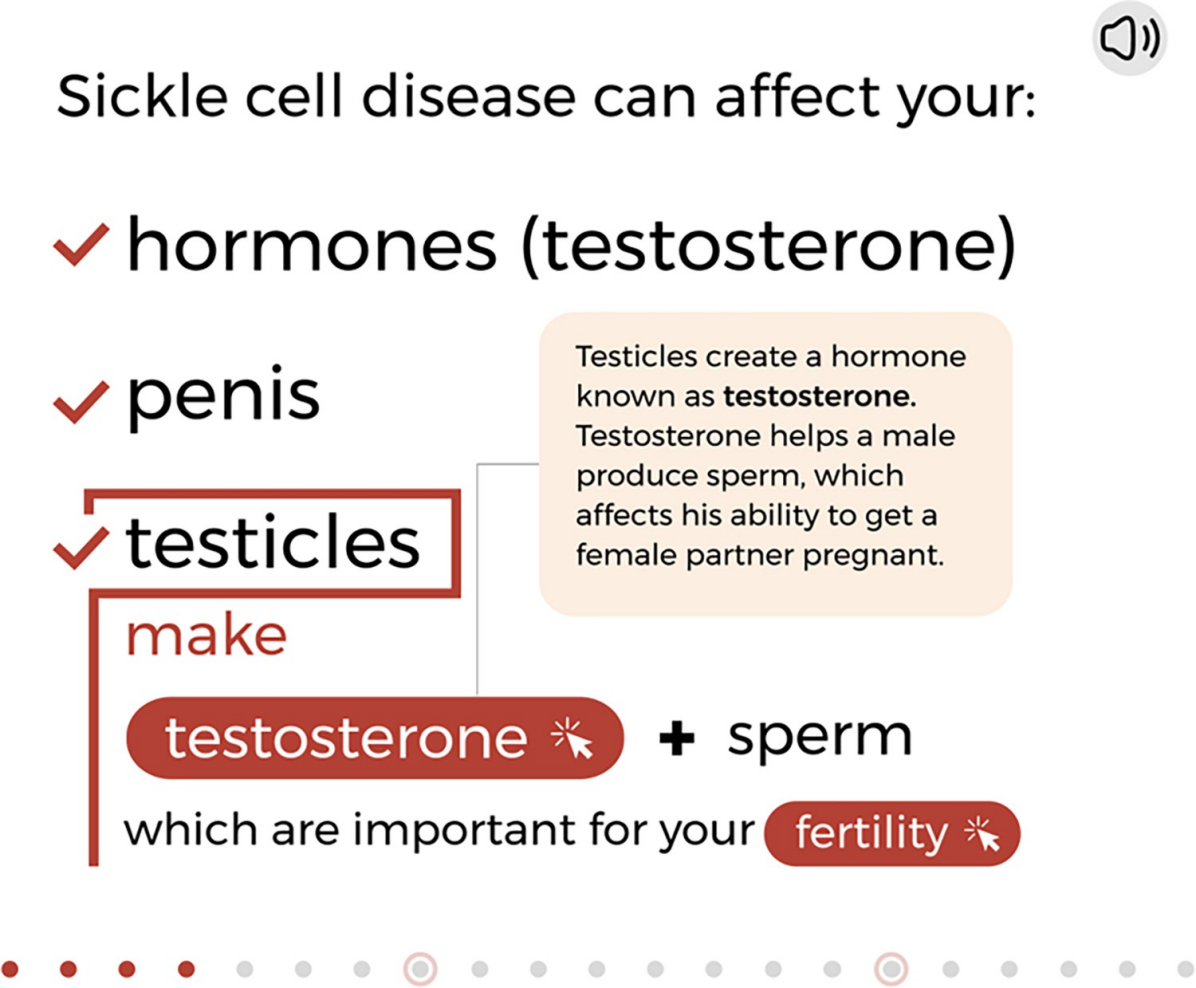
Example of an interactive slide to promoting engagement with the program. © 2023, Nationwide Children’s Hospital.

**Fig 3:**
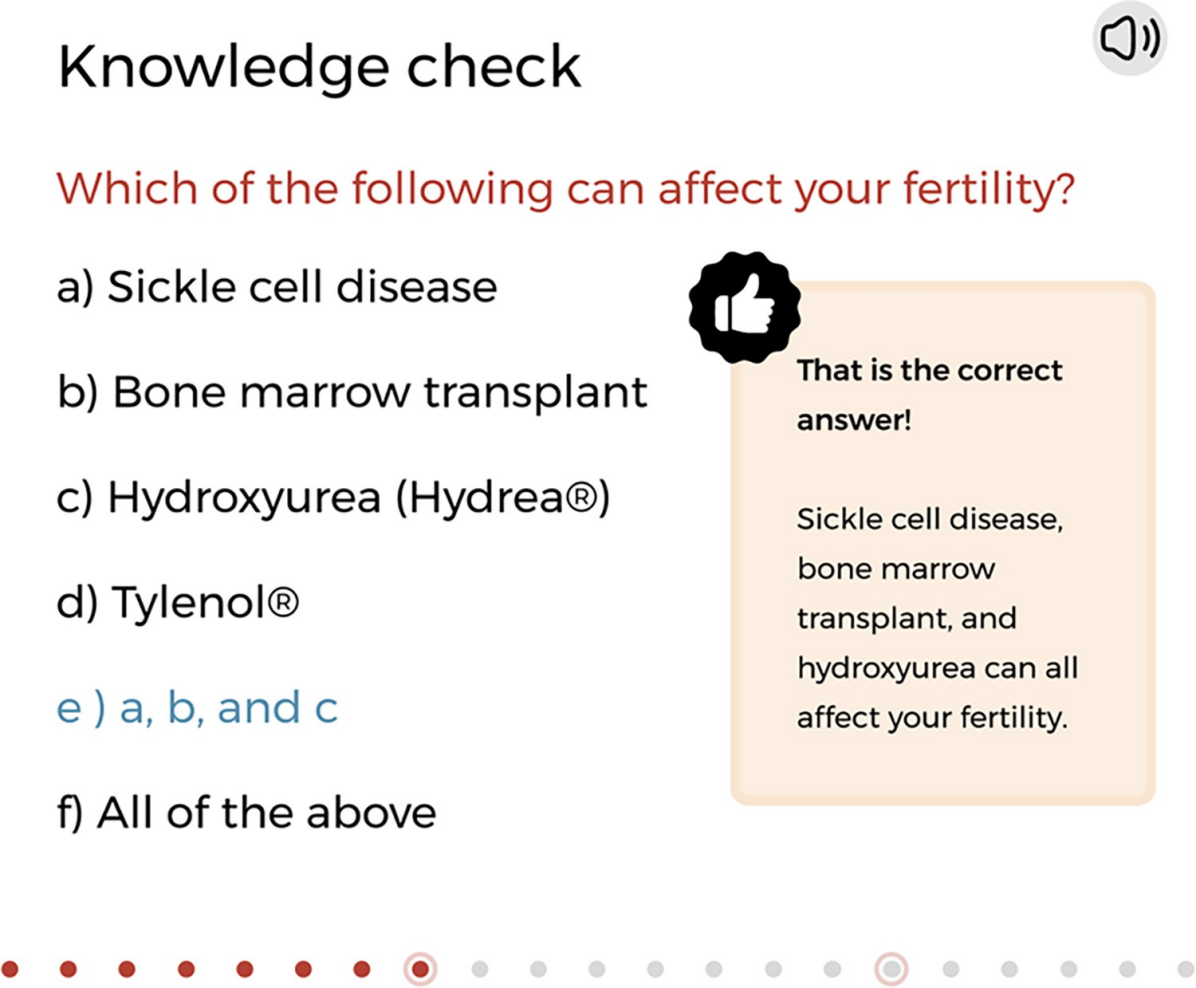
Example of a knowledge check slide to promote engagment and mastery of the topic. © 2023, Nationwide Children’s Hospital.

The design team joined the second meeting to discuss graphics and interactive qualities to include. The team decided that having a) information boxes that would appear when certain figures were clicked (Fig 2) b) knowledge checks in the forms of multiple-choice and true/false questions to promote engagement (Fig 3) and c) answer explanations (confirming or correcting answer selection) to improve retention of the material (Fig 3) were important to include. To make the program accessible, the design team chose an easy-to-use and distributable platform (Adobe™) and incorporated voice-over. Design prototypes were iteratively reviewed by all team members (i.e., research team, CAB, design team). After several rounds of refinements, the finalized FUTURES program included 27 slides, taking approximately 5–10 minutes to complete.

### Data management plan

Data collection, through REDCap, will be the responsibility of the study staff under the supervision of the principal investigator (PI). The PI will be responsible for ensuring the accuracy, completeness, legibility, and timeliness of the data reported. Clinical and laboratory data will be entered into a secure study database (REDCap). The data system includes password protection and internal quality checks, such as automatic range checks, to tag any data that appear inconsistent, incomplete, or inaccurate. Clinical data will be entered directly from the source documents. Only authorized study staff will have access to study data.

### Safety considerations

It is expected that completing the online education program and follow-up measures will be of minimal risk to participants, yet the study team acknowledges that this is a sensitive topic for many AYA and families. Participants may experience some discomfort and/or embarrassment when completing the education program and surveys, although we do not expect this to have severe or long-lasting effects on the respondent. After participating in the study, participants may have anxiety about their reproductive health and about disease modifying therapies. If so, these AYA will be referred to the psychosocial team and their hematologist who routinely addresses these concerns. Also, participants who are recruited in-person will be approached prior to their visits so that their provider can address these concerns at the visit. Loss of confidentiality is also a potential risk, although no more so than in any other research study. Participants will be notified of this potential risk.

### Data/Statistical analyses

#### Quantitative

For Phase I, frequencies will be used to describe demographics, health literacy, pre- and post-education knowledge (FKAQ), and satisfaction (acceptability) scores. Paired samples *t*-tests will be conducted to test if knowledge (e.g., FKAQ scores) significantly change after viewing FUTURES. Pearson’s correlations and chi squares will be used to investigate the relationship between demographic variables, health literacy, FKAQ scores, and satisfaction with the program. For phase II, fertility testing at a local center will be considered feasible if ≥25% of enrolled AYA in Phase I complete testing and have a viable sample; facilitators and barriers will be explored qualitatively. Additionally, descriptive analyses will be used to examine semen parameters.

#### Qualitative

Interview recordings from Phase II will be imported and transcribed in NVivo. Interview data will be analyzed by trained research staff using thematic content analysis [37]. Multiple research team members will code interview data. Kappa scores will be calculated in NVivo to determined intercoder reliability. Coders will iteratively develop tentative categories as they review interview transcripts. Interview transcripts will be reviewed in batches of five for AYA and caregivers, until saturation is reached for both groups. At this time, the research team will develop a final codebook with defined themes. Research staff will then review transcripts to determine frequency counts for each theme.

## Discussion

As AYA with SCD begin thinking about their parenthood goals, it is essential that they and their caregivers have the knowledge necessary to make informed decisions about their reproductive health (i.e., potential for disease/treatment related side-effects, information about semen parameters, testing options) [7–10]. This is especially important considering the adverse outcomes on psychosocial well-being that can occur due to missed biological parenthood opportunities [38]. As such, FUTURES aims to educate male AYA and their caregivers on the potential fertility impacts of SCD and its treatments, genetic implications of SCD, safe sex practices, and the method and purpose of fertility testing. Additionally, this study aims to explore the feasibility of fertility testing at a local testing site in this population.

FUTURES is a novel, comprehensive web-based educational program designed to address reproductive health-related knowledge gaps among male AYA with SCD and their caregivers. FUTURES was created through an iterative process involving the collaboration of clinicians, researchers, visual communication design experts, and community members affected by SCD to promote the efficacy of both content and format for those with varying levels of health literacy. Our ongoing study will assess the feasibility (recruitment rates and FUTURES video completion), acceptability (satisfaction survey), and efficacy (FKAQ outcome data) of this program among male AYA (aged 14–22 years) with SCD and their caregivers, as well as the feasibility of fertility testing (percent of participants completing testing and facilitators/barriers) for this population. While this study will be limited in scope (conducted at a single site with community advisory board members that are more engaged than the general SCD population), results will be used to inform programmatic revisions followed by larger, multi-site, implementation-effectiveness studies. Moving forward, the researchers plan to review associations between measures of healthy literacy and learning outcomes to see if additional actions need to be taken to increase knowledge. Anticipated outcomes of this line of research include improvements in reproductive health knowledge and psychosocial outcomes within this population and ultimately adaptations of FUTURES to support reproductive health care in other populations at-risk of infertility distress (e.g., female AYA with SCD and their caregivers).
